# Should Sputum Smear Examination Be Carried Out at the End of the Intensive Phase and End of Treatment in Sputum Smear Negative Pulmonary TB Patients?

**DOI:** 10.1371/journal.pone.0049238

**Published:** 2012-11-09

**Authors:** Sumit Malhotra, Sanjay P. Zodpey, Shivani Chandra, Ram Pal Vashist, Srinath Satyanaryana, Rony Zachariah, Anthony D. Harries

**Affiliations:** 1 All India Institute of Medical Sciences, New Delhi, India; 2 Public Health Education, Public Health Foundation of India, New Delhi, India; 3 State TB Office, New Delhi, India; 4 International Union Against Tuberculosis and Lung Diseases (The Union), South East Asia Regional Office, New Delhi, India; 5 Medecins sans Frontieres, Medical Department (Operational Research), Brussels Operational Center, MSF-Luxembourg, Luxembourg, Luxembourg; 6 International Union Against Tuberculosis and Lung Disease (The Union), Paris, France; 7 Department of Infectious and Tropical Diseases, London School of Hygiene and Tropical Medicine, London, United Kingdom; San Francisco General Hospital, University of California San Francisco, United States of America

## Abstract

**Background:**

The Indian guidelines on following up sputum smear-negative Pulmonary tuberculosis (PTB) patients differ from the current World Health Organization (WHO) guidelines in that the former recommends two follow up sputum examinations (once at the end of intensive phase and the other at the end of treatment) while the latter recommends only one follow up sputum smear microscopy examination, which is done at the end of the intensive phase. This study was conducted to examine if there was any added value in performing an additional sputum smear examination at the end of treatment within the context of a national TB program.

**Methods:**

This study was a descriptive record based review conducted in nine tuberculosis (TB) units in Delhi, India. All consecutive new sputum smear-negative PTB patients registered in these nine TB units from 1^st^ January 2009 to 31^st^ December 2009 were included in the study.

**Results:**

Of 2567 new sputum smear-negative TB patients, 1973 (90%) had sputum specimens examined at the end of the intensive phase, of whom 36 (2%) were smear-positive: the majority (n = 28) successfully completed treatment with either the same or a re-treatment regimen. At treatment completion, 1766 (85%) patients had sputum specimens examined, of whom 16 (0.9%) were smear-positive: all these were changed to a re-treatment regimen. Amongst the sputum-positive patients identified as a result of follow up (n = 52), four were diagnosed with multi-drug resistant TB (MDR-TB), three of whom were detected after smear examination at the end of treatment.

**Conclusions:**

Given the high burden of TB in India, a 0.9% additional yield of smear-positive sputum smears at the end of treatment translates to 3,297 cases of smear-positive PTB. End-of-treatment smear is a low-yield strategy for detection of smear-positive TB cases, although further studies are needed to determine its population-level impact and cost, particularly in relation to other TB control interventions.

## Introduction

India is the highest tuberculosis (TB) burden country in the world accounting for one fifth of global incidence [1]. In 2009, 1.5 million patients were placed on anti-TB treatment in India, of whom 366,381 (24%) were registered as new sputum smear-negative pulmonary TB cases [2].

According to current Indian guidelines, a patient is labelled as sputum smear-negative pulmonary tuberculosis (PTB) when he/she has clinical features of PTB, has two consecutive sputum smear examinations negative for acid-fast bacilli (AFB) and has radiographic abnormalities consistent with active PTB, as determined by a medical officer [3]. A patient whose sputum smears are negative for AFB but who has *Mycobacterium tuberculosis* identified on culture is also designated as sputum smear negative PTB. Such patients who are placed on treatment have follow up sputum smear examinations performed twice during the entire course of anti-TB treatment. This includes a first follow-up smear at the end of the intensive phase of treatment (2 months) and a second smear at the end of treatment (6 months) [3]. The purpose of these follow up smear examinations is to assess the response to therapy, check on whether there has been a misdiagnosis, and to determine if there is disease progression due to non-adherence or drug resistance. All these factors could result in an initially smear-negative PTB patient becoming smear-positive at the end of the intensive phase or on completion of treatment.

The Indian guidelines on following up sputum smear-negative PTB patients differ from the current World Health Organization (WHO) guidelines in that the latter recommends only one follow up sputum microscopy examination, which is done at the end of the intensive phase [4].This made us to ask; is there added value in performing an additional sputum smear examination at the end of treatment within the context of a national TB program?

In a cohort of newly registered sputum smear-negative PTB patients, we determined i) the proportion of patients who had sputum smear examinations done at the end of the intensive phase and at the end of treatment, ii) the number and percentage of patients with positive sputum smears at these time points and iii) the treatment outcomes of those patients who were found to be sputum smear-positive.

## Materials and Methods

### Design

This was a descriptive study using routine programme data.

### Study setting

The study was conducted in the state of Delhi, the National Capital of India. Delhi has an area of 1483 sq km, with a total population of 17 million and population density of 11,000/sq km, with 40% of the population living in slums or similar contexts. The Revised National TB Control Programme (RNTCP) has been implemented in all districts of Delhi since 1997. For the management of RNTCP, the state has been divided into 24 Chest Clinics [5]. Under each Chest Clinic, there is one Tuberculosis Unit (TU) for half a million population having a Designated Microscopy Centre for every 0.1 million population. The 24 Chest Clinics under RNTCP fall within the nine Revenue Districts of Delhi State.

Patients were registered in the programme in one of three treatment categories: details of these categories and the treatment regimens are presented in **Table 1**. At the end of treatment, one of several mutually exclusive treatment outcomes is given to patients as shown in **Table 2**. Most of the new sputum smear-negative pulmonary TB patients were treated with a category 3 regimen, but some with serious illness were treated with a category 1 regimen. The patients were treated using the Directly Observed Treatment Short course (DOTS) approach, and were supervised regularly thrice a week in the intensive phase and once a week during the continuation phase. They were followed with sputum smear examination at the end of the intensive phase (2 months) and on treatment completion (6 months) [3]. Sputum smears were examined in quality assured laboratories and results were recorded in the TB register. If patients failed on treatment (i.e., were found to be sputum smear positive at 5 months or later), they were changed to a retreatment category 2 regimen. Such patients were also considered to be suspects of MDR-TB (Multi-Drug Resistant TB-resistant to rifampicin and isoniazid) and their sputum specimens were sent to an accredited laboratory facility for culture and drug sensitivity testing.

**Table 1:** Treatment categories and regimens under Revised National TB programme, India.

Treatment category	Type of patients	Treatment regimens***	
		Intensive Phase	Continuation phase
Category 1	New sputum smear-positive PTB	2(H_3_R_3_Z_3_E_3_)	4(H_3_R_3_)
	New sputum smear-negative PTB, seriously ill*		
	New extra-PTB, seriously ill*		
Category 2	Sputum smear-positive relapse	2(H_3_R_3_Z_3_E_3_S_3_)+1(H_3_R_3_Z_3_E_3_)	5(H_3_R_3_E_3_)
	Sputum smear-positive treatment failure		
	Sputum smear-positive treatment after default		
Category 3	New sputum smear-negative, not seriously ill**	2(H_3_R_3_Z_3_)	4(H_3_R_3_)
	New extra-PTB, not seriously ill**		
Category 4	Multi Drug Resistant –TB (MDR-TB)****	6 (9) Km Lvx Eto Cs Z E (daily)	18 Lvx Eto Cs E (daily)

PTB = Pulmonary Tuberculosis; EPTB = Extrapulmonary Tuberculosis; H = Isoniazid; R = Rifampicin; Z = Pyrazinamide; E = Ethambutol; Km = Kanamicin; Lvx = Levofloxacin; Eto = Ethionamide; Cs = Cycloserine.

The number before the letters indicates number of months of treatment; numbers in subscript indicate the number of days per week of doses.

* Seriously ill includes patients that have military TB, extensive parenchymal infiltration, co-infection with HIV, cavitary disease. In children, seriously ill sputum smear-negative PTB includes all forms of sputum smear-negative PTB other than primary complex. Seriously ill EP-TB includes TB meningitis (TBM), disseminated TB, TB pericarditis, TB peritonitis and intestinal TB, bilateral extensive pleurisy, spinal TB with or without neurological complications, genitourinary TB, and bone and joint TB.

** Not seriously ill sputum smear-negative PTB includes primary complex. Not seriously ill EP-TB includes lymph node TB and unilateral pleural effusion.

*** The dosage strengths are as follows: H : Isoniazid (600 mg), R:Rifampicin (450 mg), Z: Pyrazinamide (1500 mg); E: Ethambutol (1200 mg); S: Streptomycin (750 mg).

**** MDR-TB diagnosis is done through culture and drug sensitivity testing from a quality-assured laboratory. Treatment of MDR-TB uses a standardized regimen, comprising of 6 drugs – Km (Kanamycin), Lvx (levofloxacin), Eto (Ethionamide), Z (pyrazinamide), E (Ethambutol) and Cs (Cycloserine) during 6–9 months of intensive phase and 4 drugs (Lvx, Eto, E and Cs) during the 18 months of continuation phase. Dosages of drugs are based on three weight bands (16–25 kg, 26–45 Kg, and >45 Kg).

**Table 2:** Definitions of treatment outcomes.

**Outcome: Definition**
**Cured**: A patient who is initially sputum smear-positive, who has completed treatment and has negative sputum smears, on two occasions, one of which was at the end of treatment.
**Treatment completed**: A patient who is sputum smear-positive who has completed treatment, with negative smears at the end of the intensive phase but none at the end of treatment.
Or: A sputum smear-negative TB patient who has received a full course of treatment and has not become smear-positive during or at the end of treatment.
Or: An extra-pulmonary TB patient who has received a full course of treatment and has not become smear-positive during or at the end of treatment.
**Treatment success**: Includes cured and treatment completed together
**Death**: A patient who died during the course of treatment regardless of cause
**Failure**: A TB patient who is smear positive at 5 months or more after starting treatment. Failure also includes a patient who was treated with Category III regimen but who becomes smear positive during treatment. And the patient is not put on MDR-TB treatment
**Defaulted**: A patient who has not taken anti-TB drugs for 2 or more consecutive months after starting treatment.
**Transferred out**: A patient who has been transferred to another Tuberculosis Unit/District and his/her treatment outcome is not known
**Switched over to MDR-TB treatment**: A patient who has been diagnosed as having MDR-TB by an RNTCP accredited laboratory, prior to being declared as “Failure”, and is placed on the RNTCP MDR-TB treatment regimen

### Study population

From each of the nine revenue districts of Delhi, if there were more than one tuberculosis units, the TB unit that registered the maximum number of patients during the study period was selected for inclusion in the study. All consecutive new sputum smear-negative pulmonary TB patients registered in these nine TB units from 1^st^ January 2009 to 31^st^ December 2009 were included in the study.

### Data collection and analysis

Data were obtained from the TB registers. A data extraction sheet was used by the study investigators to capture details on sputum smear examination and treatment outcome status at the end of the intensive phase and on treatment completion. Cross validation of the records was undertaken for 10% of smear-negative Pulmonary TB patients by examining patient treatment cards and TB Laboratory registers. Data were entered and analyzed using a Microsoft Excel spreadsheet Version 2007.

### Ethics approval

Ethical approval was obtained from the Institutional Ethics Committee of Public Health Foundation of India, New Delhi and the Union Ethics Advisory Group, Paris. The study was a retrospective review of routine programme records and reports, and permission was obtained from the programme managers at the state and national levels to access these data. Individual patient consent was deemed un-necessary by both the ethics committees. Electronic databases created for the analysis were stripped of personal health identifiers and maintained securely.

## Results

### Sputum examinations during and at end of treatment

**Figure 1:**
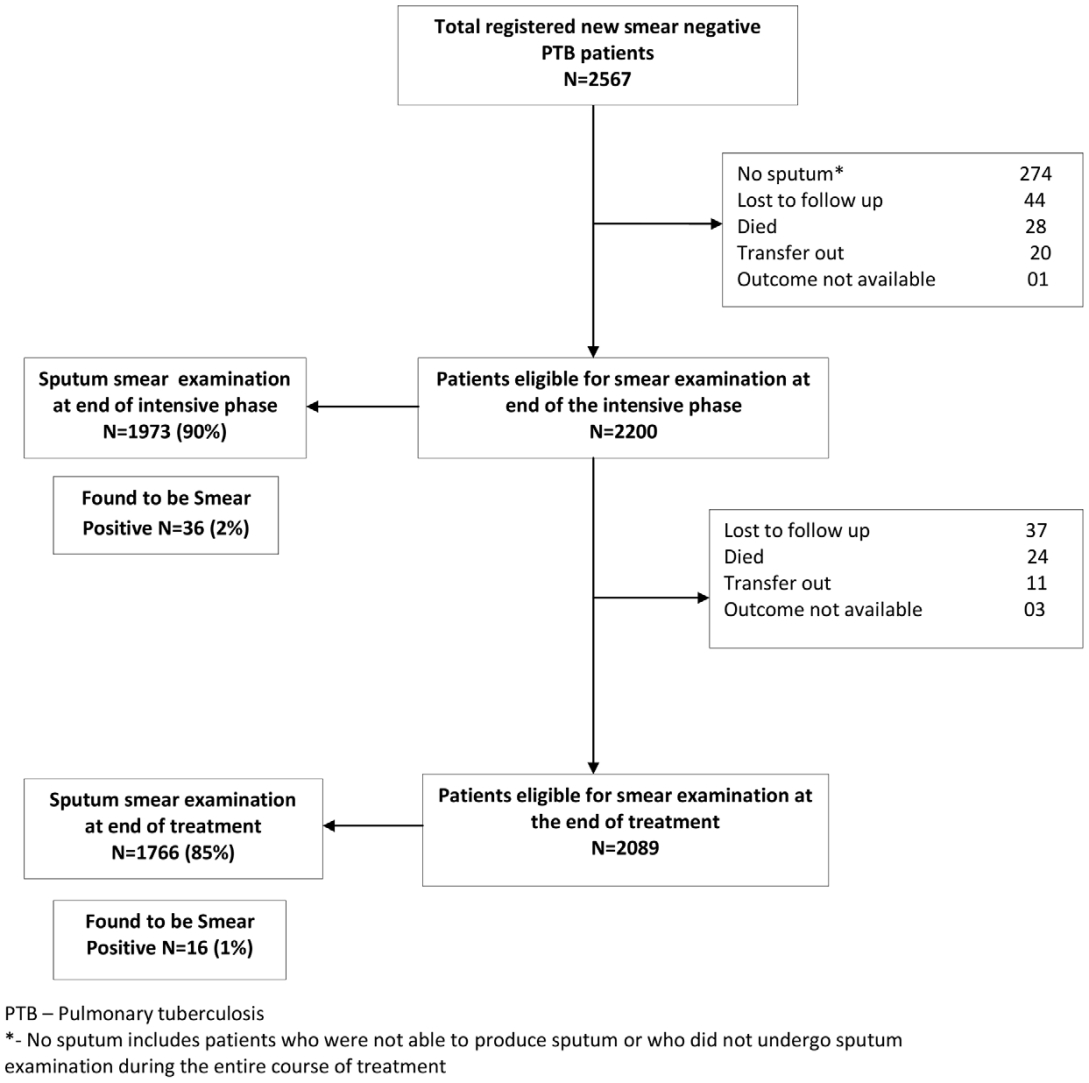
Flow Chart showing sputum smear examination at end of intensive phase and end of treatment in new smear negative pulmonary tuberculosis patients in Delhi, India (2009).

A total of 2,567 new sputum smear negative PTB patients were registered in the 9 TB units in Delhi. The management of these patients and the numbers who were eligible for and had sputum smear examination performed from diagnosis until the end of treatment is shown in **Figure 1**.

### Smear status at the end of the Intensive phase of anti-tuberculosis treatment

**Figure 2:**
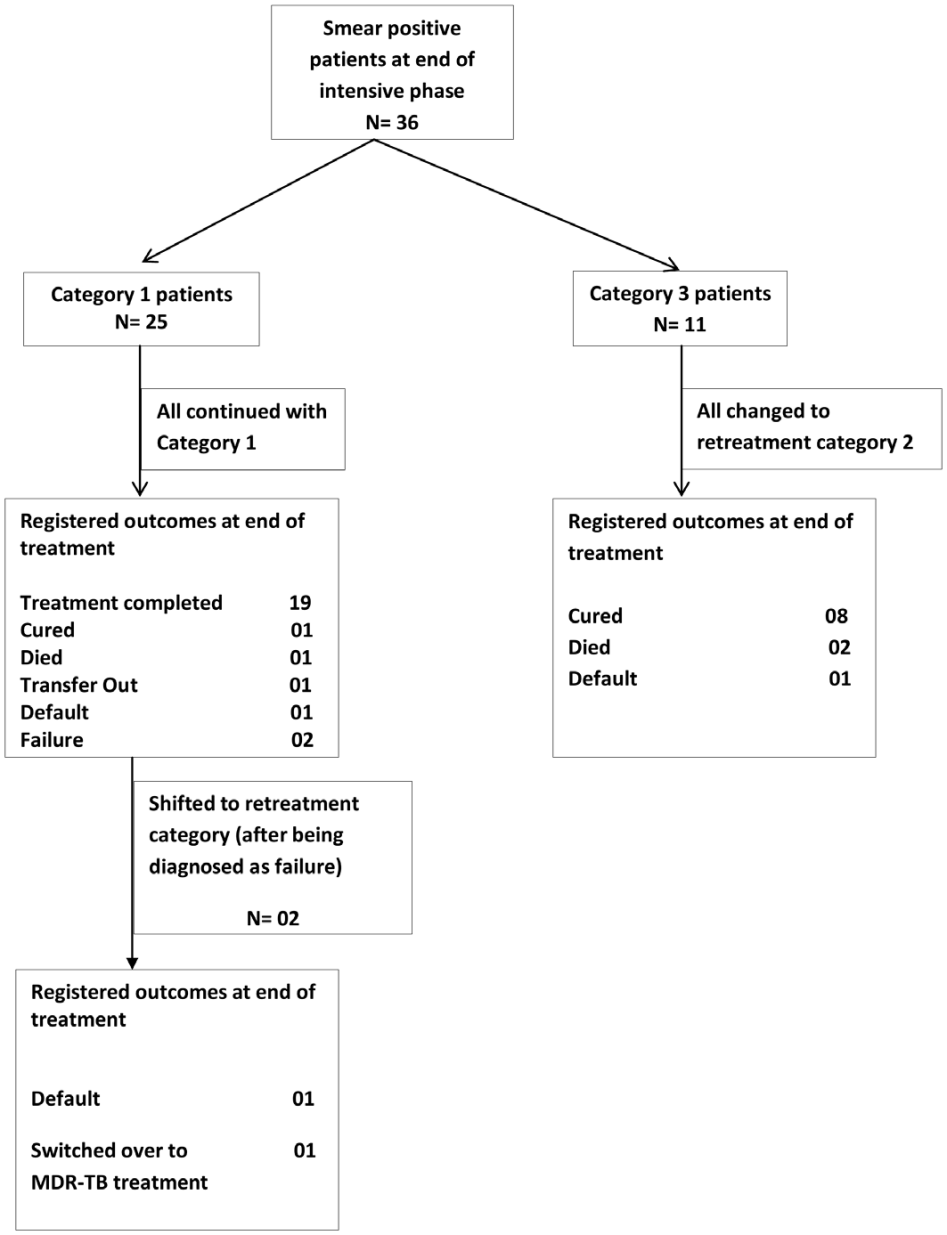
Final treatment outcomes in patients who were smear-positive at the end of the intensive phase of anti-tuberculosis treatment.

Of all newly registered patients, 367 (14%) were not eligible for sputum smear examination at end of the intensive phase due to various reasons shown in **Figure 1**, Of those eligible for sputum smear examination at this point, 227 (10%) failed to have the examination. There were 1973 patients whose sputum smears were examined, of whom 36 (2%) were sputum smear-positive. Of the latter, 25 were receiving category 1 treatment and 11 receiving category 3 treatment. The treatment outcomes of patients who were smear-positive at the end of the intensive phase are shown in **Figure 2**. Amongst the category 1 patients who were found to be smear-positive (25), all continued their same treatment regimens and the majority successfully completed treatment. There were two patients who were changed to a retreatment category 2 regimen, of whom one was later diagnosed with multi-drug resistant TB (MDR-TB, i.e., TB resistant to both isoniazid and rifampicin) and switched over to category 4 treatment for its management. Of the patients who were receiving category 3 treatments and were smear-positive at the end of the intensive phase (11), all were changed to a re-treatment category 2 regimen. The majority of these patients had a successful treatment outcome.

### Smear status at the end of anti-tuberculosis treatment

During the continuation phase of treatment, 111 (5%) patients were not eligible for sputum smear examination at the end of treatment (6 months) due to various reasons shown in **Figure 1**. Of those eligible for sputum smear examination, 323 (15%) did not have the examination done. There were 1766 patients whose sputum smears were examined at the end of treatment, of whom 16 (0.9%) were sputum smear-positive.

**Figure 3:**
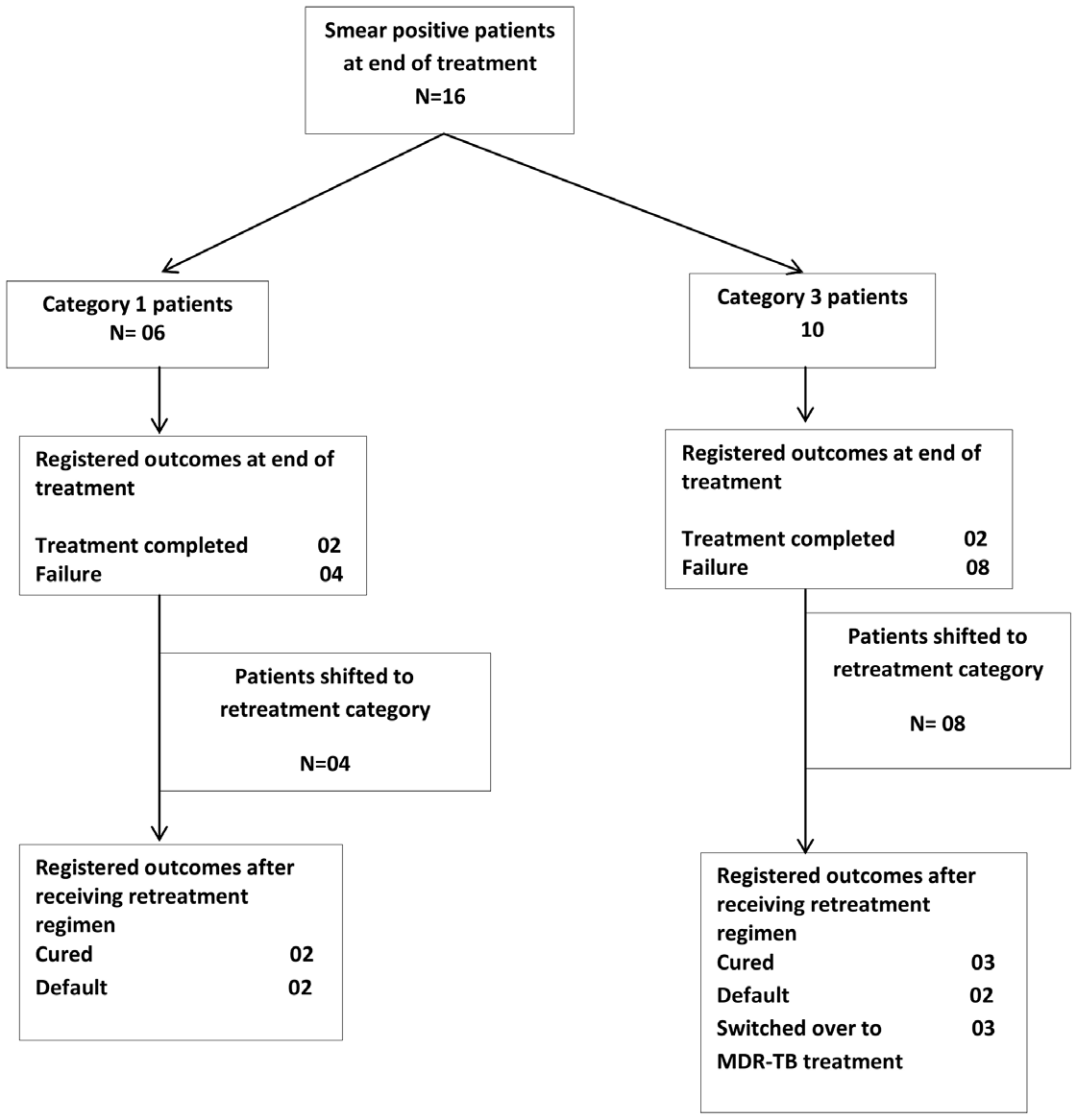
Final treatment outcomes in patients who were smear-positive at the end of anti-tuberculosis treatment.

Amongst these smear-positive patients, 6 were receiving category 1 and 10 were receiving category 3 treatment regimens. Of the 16 smear-positive patients, 12 were changed to a category 2 retreatment regimen. Two other patients from each category were lost to follow up after their treatment was completed and their outcome was registered as treatment completed. The outcomes of the 12 patients who were changed to a retreatment regimens shown in **Figure 3**. Three of these 12 patients were later diagnosed with MDR-TB and were switched to a MDR-TB treatment regimen for its management.

### Sensitivity analysis and costs for sputum smear at end of anti-tuberculosis treatment

A sensitivity analysis was performed to check what the estimate would have been if all of the patients who did not get their last follow up sputum smear examination turned out to be smear-positive or smear-negative. If all the missing patients had been smear-positive, the smear-positivity rate at the end of treatment would have been 16%. Conversely, if all these patients had been smear-negative, then the estimate would have been 0.8%. Even with the low estimate of 0.8%, from a public health perspective, the absolute number in terms of cases of potentially resistant TB cases identified is still significant.

The cost of undertaking a single sputum smear examination in our setting is US $ 0.90 [6]. In this study, 1766 patients at the end underwent sputum examination with 15 patients experiencing a change in either treatment category, yielding a Cost Benefit Ratio (CBR) of US $ 106/ treatment regimen changed. If all eligible patients in the study cohort (2089) had undergone the last sputum smear examination and all the missing patients were smear positive resulting in a change in treatment regimen, the CBR would have been US $ 5/ treatment regimen changed. On the other hand, if all the missing patients were smear-negative, CBR would have been US $ 125/ treatment regimen changed.

## Discussion

This is one of the first studies evaluating the added value of sputum smear examinations at the end of the intensive as well as the end of treatment in new sputum smear negative PTB patients. The majority of patients who were eligible to submit sputum specimens at the end of the intensive phase of treatment and at the end of treatment had their sputum smears examined. Smear-positivity was 2% for the smear examination at the end of the intensive phase and 0.9% at the end of treatment. These percentages may seem low, but in India with an estimated 366,381 annual new smear negative PTB patients registered in 2009, this translates to over 7000 cases with positive smears at 2 months and 3297 cases of smear positive PTB at the end of treatment. For patients on the category 3 regimen, all of those with smear-positive sputum at 2 months were changed to a retreatment regimen with a generally successful treatment outcome, so action was taken on the result. Action was not so clear for those found to be smear-positive at the end of treatment. A quarter of patients were incorrectly registered as treatment completed. The remainder was changed to a re-treatment regimen with mixed results, but three patients were identified with MDR-TB and changed to the appropriate treatment, an action which would not have occurred if the smear examination had not been performed.

There are important public health implications from these findings. First, persistent smear positivity at the end of treatment completion might herald misdiagnosis, failure on current drug regimens or primary or acquired drug resistance. In particular, the possibility of persistent smear positivity heralding MDR-TB is of major concern as this form of drug resistance needs early identification in order to limit its spread to households and the community at large. Such cases will add to the “difficult to manage” TB cases and the already high background burden of disease. Second, from a patient perspective, this failure of treatment is likely to impact on individual survival. It is not known what happened to the patients with smear-positive sputum at the end of treatment who were incorrectly declared successfully treated or what happens in general to these patients if the smear examination is not performed. However, it is likely that outcomes are compromised.

One consideration is that our study group included patients in category 3 with a three-drug treatment regimen in the intensive phase in addition to patients in category 1 who were treated with a four-drug treatment regimen in the intensive phase. The category 3 regimen is considered a relatively inferior regimen and no longer exists in the country. It is possible that the country-wide shift to a category 1 regimen for all smear-negative PTB patients might affect our study estimate of 1%. It is thus urgent that RNTCP audits current data under the revised national guidelines to fully validate these findings.

The strengths of this study are that a large number of patients were studied, and we used routine programme data. The findings are therefore likely to reflect the operational reality on the ground. We have also adhered to the STROBE guidelines on reporting [7].

The study has a number of limitations. First, the use of record reviews does mean that sometimes the data are inaccurately recorded. Second, our estimate of the added value of 0.9% positive sputum smears at the end of treatment might be affected by the 15% of patients who for various reasons did not undergo the last follow up smear examination at the end of treatment completion. A large number of patients not undergoing follow-up sputum smear examination is not unique to this study and/or region and has also been highlighted in another study from a different region in the country [8]. Even with a low estimate of 0.8%, obtained through the sensitivity analysis presented in the results, from a public health perspective, the absolute number in terms of cases of potentially resistant TB cases identified is still significant. Third, sputum smear microscopy as a tool of diagnosis is challenged by the issue of false positive and false negative results, which may occur even within quality assured programmes. However, sputum smear microscopy is the mainstay of diagnosis programmatically in India, and is used to identify patients who may be at risk of drug-resistant disease during follow up and who may need additional culture and drug sensitivity testing. Finally, we were unable to ascertain the number of patients who were sputum smear positive in follow up smears and were concomitantly HIV-seropositive as during the study period HIV testing was not routinely performed.

This is the first report of its kind coming from a high TB burden country like India, but like many studies it raises additional questions. First, the RNTCP has stopped giving category 3 treatment, and therefore a similar study with the same research question should be conducted for another cohort of patients receiving the changed treatment regimens. Second, a prospective study should be done for a similar cohort of patients as this would obviate a number of the limitations inherent in this retrospective record review study. A prospective study could also include sputum culture and drug sensitivity testing on all specimens as well as a detailed cost-analysis on all aspects. The decision to continue sputum smear examination at end of continuation phase will also be dependent on CBR of the additional sputum smear examination. Given the political commitment by the Government of India to support the RNTCP with the aim of improving its performance over several years, the current costs of performing the additional end-of-treatment smear as presented in the results section, do not seem to impose an economic challenge to the programme. The end of smear examination in patients with smear-negative pulmonary TB must be assessed in relation to other strategies for improving case detection as part of the country's TB control efforts; for example, comparisons made with contact investigation of index patients where the yield may be in the region of 2–5% [9].

In light of above and the evidence generated through this study, there is need for further evaluation on this research question. End-of-treatment smear is a low-yield strategy for detection of smear-positive TB cases, although further studies are needed to determine its population-level impact and cost, particularly in relation to other TB control interventions.
